# How Much Support Is There for the Recommendations Made to the General Population during Confinement? A Study during the First Three Days of the COVID–19 Quarantine in Spain

**DOI:** 10.3390/ijerph17124382

**Published:** 2020-06-18

**Authors:** Carlos Suso-Ribera, Ramón Martín-Brufau

**Affiliations:** 1 Department of Basic and Clinical Psychology and Psychobiology, Jaume I University, 12071 Castellon de la Plana, Spain; 2 Murcia Health Service, 30800 Murcia, Spain; r.martinbrufau@um.es; 3 Department of Personality, Assessment, and Psychological Treatments, Murcia University, 30100 Murcia, Spain

**Keywords:** COVID–19, mood states, quarantine, lifestyle, frustration intolerance

## Abstract

*Background:* Recommendations on lifestyles during quarantine have been proposed by researchers and institutions since the COVID–19 crisis emerged. However, most of these have never been tested under real quarantine situations or derive from older investigations conducted mostly in China and Canada in the face of infections other than COVID–19. The present study aimed at exploring the relationship between a comprehensive set of recommended lifestyles, socio–demographic, and personality variables and mood during the first stages of quarantine. *Methods*: A virtual snow–ball recollection technique was used to disseminate the survey across the general population in Spain starting the first day of mandatory quarantine (15 March 2020) until three days later (17 March). In total, 2683 Spanish adults (mean age = 34.86 years, *SD* = 13.74 years; 77.7% women) from the general population completed measures on socio–demographic, COVID–related, behavioral, personality/cognitive, and mood characteristics. *Results*: In the present study, depression and anger were higher than levels reported in a previous investigation before the COVID–19 crisis, while vigor, friendliness, and fatigue were lower. Anxiety levels were comparable. The expected direction of associations was confirmed for the majority of predictors. However, effect sizes were generally small and only a subset of them correlated to most outcomes. Intolerance of unpleasant emotions, neuroticism, and, to a lesser extent, agreeableness, sleep quality, young age, and time spent Internet surfing were the most robust and strongest correlates of mood states. *Conclusions*: Some recommended lifestyles (i.e., maintaining good quality of sleep and reducing Internet surfing) might be more important than others during the first days of quarantine. Promoting tolerance to unpleasant emotions (e.g., through online, self–managed programs) might also be of upmost importance. So far, recommendations have been made in general, but certain subgroups (e.g., certain personality profiles and young adults) might be especially vulnerable and should receive more attention.

## 1. Introduction

The World Health Organization (WHO) declared, in January 2020, the outbreak of COVID–19, a new coronavirus disease. The WHO indicated that there was an elevated risk of coronavirus spreading globally and, indeed, in March 2020 the situation was characterized as a pandemic [1]. In Spain, the Government declared the state of alarm late on 14 March 2020. As a consequence, since March 15 an absolute quarantine was imposed and individuals were only allowed to leave their homes under certain circumstances, such as to buy medicines, food, or travel to health centers. This also implied closing all non–essential services and stores, including restaurants [2]. 

It has been recently argued that quarantine will boost mental health problems in the population and several authors have anticipated that anxiety, depression, and anger will be frequent [3,4]. Thus, since quarantine and mobility restrictions have become more and more frequent globally, institutions, researchers, and clinicians have made an effort to provide the population with guidelines on how to manage quarantine in a more effective manner for their mental well–being. An example of the previous was a rapid review published in *The Lancet* as early as March 14, in which past research on the psychological consequences of quarantine due to similar circumstances (e.g., severe acute respiratory syndrome, influenza, Ebola, and Middle East respiratory syndrome–related coronavirus) and their correlates were investigated [5]. The authors concluded that quarantine has important psychological effects on individuals and pointed to a number of stressors, such as mobility restriction duration, fear of infection, frustration, boredom, inadequate supplies, and inadequate information. However, the cross–cultural generalizability of the findings and applicability to the COVID–19 situation was put into question because previous sample sizes were generally small, the majority of studies had been conducted in Canada and China, and many only included specific populations (i.e., students or health–care workers). 

While acknowledging the aforementioned stressors, many local and global entities and agencies have provided their own psychological recommendations to better manage the quarantine. For instance, in March 18, the WHO addressed a message to the population and suggested some best practices, such as minimizing seeking news about COVID–19 that increased anxiety or distress and being supportive to others (e.g., checking by telephone on people in the community) [1]. Other entities, such as the Centers for Disease Control and Prevention, the American Psychiatric Association, the National Health Service, and Spanish institutions (Spanish Government and General Council of the Official College of Psychologists), to name some examples, have made similar efforts [6,7,8,9]. In general, these guidelines have recommended limiting COVID–19 news exposure, taking care of the body (e.g., exercising, eating healthy and at regular times, getting enough sleep, avoiding drug and alcohol use), planning a daily routine, getting involved into pleasant activities, and connecting with trusted others to share concerns and feelings, which is consistent with recent opinions from researchers in the field [10,11,12]. To what extent are these and other recommendations that have been frequently posted in the media associated with a successful adaptation to quarantine in the current COVID–19 crisis? 

Psychological interventions in the face of the COVID–19 crisis should be based on sound scientific advice [13]. However, current evidence is insufficient to confirm to what extent the recommended lifestyles reviewed in the previous paragraphs are associated with a more successful adaptation to the quarantine in the present pandemic. Additionally, because research in the field has been conducted in China, the cross–cultural generalizability of findings is unclear. Therefore, the goal of the present study is to investigate the emotional impact of the COVID–19 pandemic, as well as to the extent to which a comprehensive set of socio–demographic, behavioral, and personality/cognitive correlate with well–being just at the beginning of the quarantine in Spain (first three days). According to the reviewed literature, we expect to find low mood as a result of the first days of quarantine. We also hypothesize that we will replicate most of the proposed correlates of well–being indicated in guidelines, expert opinions, and the scarce literature from China.

## 2. Materials and Methods 

### 2.1. Study Design and Participants

This is a cross–sectional, observational investigation with 2683 participants who completed an online Qualtrics^©^ survey disseminated using a virtual snow–ball sampling approach with different social media resources (e.g., Twitter, Whatsapp, Instagram, and Facebook). Specifically, social media accounts were used to disseminate the study online and respondents were asked to disseminate the study in their own social media. This is a non–probabilistic sampling strategy that can help increase the number of responses and the representativeness of the sample, yet with some bias (e.g., only people who have access to the Internet can respond and certain online populations might be more likely to participate). Survey dissemination started late in the evening on 15 March 2020, the day the quarantine officially started in Spain (even though it was officially announced the day before). This means that the population in Spain knew about the quarantine onset already on 14 March. Responses were obtained during the first three days of quarantine: 15 March (n = 713), 16 March (n = 1332), and 17 March (n = 638). The fact that data was obtained during three consecutive days should not be interpreted as this being longitudinal data. Data represents responses from different individuals and is the study is therefore cross–sectional. Data was obtained from all 17 regions in Spain. The exact details are not provided for readability reasons, but the proportion of responses per region was consistent with the population in that region (e.g., the majority of responses, that is 58.6%, were obtained from the four most populated regions in Spain, which represent 57.8% of the adult population in the country).

All obtained responses were anonymous and no personal data was collected from any of the participants. The informed consent was obtained for all participants in the same online platform, Qualtrics^©^, before allowing participants to complete the survey. First, the online survey showed an information sheet for the study and then informed consent had to be provided by clicking on a button where they confirmed that they were over 18 years of age and were willing to voluntarily participate in the investigation. The study and its procedures were approved by the ethics committee of the University of Murcia. The study was conducted in accordance with the Declaration of Helsinki. Eligibility included being over 18 years of age, understanding Spanish, living in Spain, and consenting to participate in the online consent form.

### 2.2. Measures

The primary outcome was mood, which was measured with the reduced 30–item version of the Profile of Mood States [14]. This questionnaire evaluates six dimensions of mood, namely depression, anxiety/tension, anger, vigor, fatigue, and friendliness.

The selection of predictors was based on the literature review and the recommended lifestyles from several institutions described in the previous paragraphs. These included socio–demographic and clinical characteristics, quarantine–related behavior, and personality/cognitive factors (see Table 1 and Table 2 for details).

Socio–demographic, COVID–related, and behavioral factors were created *ad hoc* and were dichotomous, ordinal, or continuous, depending on the content assessed. All behavioral variables referred to behavior in the past 24 h. For the measurement of frustration intolerance, the 7–item Emotional Intolerance scale from the Frustration Discomfort Scale was used because this is the scale that mostly correlates to negative mood states [15]. For personality, the Ten Item Personality Inventory was selected because it is a valid and very brief measure of the Big Five personality dimensions [16]. High scores in these scales should be interpreted as indicating poorer tolerance to unpleasant emotions/cognitions and higher neuroticism, extraversion, openness to experience, agreeableness, and conscientiousness. 

### 2.3. Data Analysis

Following a descriptive analysis of items/scales, the levels of the main outcome (mood) will be compared against previous scores from the general population in Spain obtained before the COVID–19 epidemic [17]. An independent *t*–test was computed and the Cohen’s *d* estimate was calculated as a measure of effect size.

Finally, a set of linear regressions was calculated for each predictor and each outcome separately to obtain a measure of the strength of the relationship between every predictor with an outcome (Table 3). Similar to recent research [18], univariate regressions are presented first to show the relationship between each predictor and outcomes. Additionally, though, we also propose an additional multivariate model that can help identify the more relevant predictors for each outcome. Due to the large number of predictors and the risk of multicollinearity problems, a back-elimination procedure was selected in the multivariate regression. In this type of regression, all variables are entered in a first step and then variables are sequentially removed based on partial correlation. This provides a parsimonious model based on the data, but not on theory. 

Linear regressions assume that the relationship between the predictor and the outcome is linear. Thus, we first tested whether this type of relationship was feasible for the ordinal variables. We calculated a series of analyses of variance (ANOVAs) and investigated whether mean scores in outcomes across categories in the ordinal predictors suggested a linear relationship between both variables. This was the case for all predictors, except for “Exercising frequency”. The ANOVA findings suggested that those that exercised were generally in a better mood, irrespective of the amount of exercise they performed. Therefore, a dichotomous version of this variable, which was labeled as “Exercised”, was created.

Because the administration of measures was completely online, we checked the time spent to complete the survey and included several control items to control for fake or inconsistent responding to the survey (i.e., “Select option *Never true* in the current item”).

Due to the large number of analyses made and to reduce the risk of false positives and unimportant associations, the alpha level was corrected for multiple testing and set to 0.001.

## 3. Results

In total, 3608 respondents consented to participate into the study after reading the online information sheet. Of these, 653 were eliminated because they did not provide any information and 272 were removed because they completed less than half of the survey, which did not include the mood questionnaire. Thus, the final sample comprised 2683 respondents. An analysis of the control items and the time spent completing the survey did not suggest the need to eliminate further participants from the sample.

### 3.1. Characteristics of the Sample

The results of the descriptive analyses are either presented in Table 1 and Table 2 (behavioral/coping) or described in the following lines for readability reasons. The average age of participants was 34.86 years (*SD* = 13.74, range = 18 to 80). The majority of them were women (77.7%). At the time of assessment, most respondents were in a relationship (76.4%) and had completed postsecondary education studies (63.5%). A significant number of individuals indicated having lost their job due to the COVID–19 pandemic (23.7%), while 45.8% of respondents reported working at the time of assessment. The representation of students in the sample was high (27.7%). Only a small percentage of participants did not cohabitate at the time of assessment (8.8%) and cohabitation with an infected person was even more infrequent (0.8%). The majority of participants did not cohabitate with children (28.9%). House size and income were relatively similarly distributed across the sample. 

Monthly home income was very diverse and ranged from less than 1000€ (8.8%) to more than 3500€ (17.6%). The percentage of individuals reporting a monthly income of 1001€–1500€, 1501€–2000€, 2001€–2500€, and 2501€–3000€ was 17.3%, 17.4%, 12.6%, and 9.4%, respectively. House sizes also largely varies across participants, with some living in homes of ≤ 70 m^2^ (21.0%) or 71–90 m^2^ (22.3%) and other living in houses of 91–110 m^2^ (23.9%), 111–130 m^2^ (13.3%), or >130 m^2^ (19.5%). 

The majority of respondents perceived that they had a moderately or extremely low risk of COVID–19 infection (68.0%) and indicated that they did not have a relative that would be considered as vulnerable to the COVID–19, such as patients with immunosuppression, cancer, or severe respiratory conditions (74.3%). Only 1.8% of participants reported taking psychotropic medication at the time of assessment.

More than half of participants reported having changed their usual sleeping patterns due to the quarantine (54.9%). However, the majority of participants were somewhat–to–completely satisfied with the quality of their sleep in the previous night (67.5%). Participants reported having slept an average of 7.31 h (*SD* = 1.25) the previous night.

Participants interacted with an average of 2.40 individuals (*SD* = 1.78) in person and 1.71 individuals (*SD* = 1.95) using videoconferences daily. Certain activities (i.e., sitting/laying, text messaging, Internet surfing, seeking COVID–19 information, and watching TV/series/movies) were very frequent and were only never performed by less than 6% of respondents. Other behaviors, such as exposing to sunlight (none = 19.0%) and exercising (none = 48.4%) were less common. When trying to cope with emotional distress and boredom, the most frequent activities were eating (not at all = 1.5%), watching TV (not at all = 11.4%), and cleaning/tidying (not at all = 13.5%). Working/studying (not at all = 38.3%), videoconferencing (not at all = 39.4%), playing games with others (not at all = 51.3%), and exercising (not at all = 56.6%) were less frequently used as coping strategies. Almost half of participants did not plan or only slightly planned their daily activities in advance (49.4%). Up to 48.4% of respondents did not exercise at all during the past 24 h. See Table 1 and Table 2 for more details on behavioral/coping items and responses.

### 3.2. Mood Status during the Beginning of the Quarantine (First Three Days) and Comparison with Data from the General Population Before the Quarantine

As indicated in Table 3, participants in the present study reported more depressed mood (*t* = 5.79, *p* < 0.001, *d* = 0.30) and anger (*t* = 4.81, *p* < 0.001, *d* = 0.25), as well as less vigor (*t* = −12.49, *p* < 0.001, *d* = 0.69) and friendliness (*t* = −9.41, *p* < 0.001, *d* = 0.47) compared to data from the general population previous to the current COVID–19 crisis [17]. By contrast, the fatigue levels were lower in the present study sample (*t* = −5.75, *p* < 0.001, *d* = 0.34). Anxiety levels were comparable across samples (*t* = −0.29, *p* = 0.774, *d* = 0.02).

### 3.3. Correlates of Mood Status

The results of the univariate regression analyses are reported in Table 4. Some variables were significantly associated with all or almost all (four out of six) outcomes. These included younger age (3.9 ≤ *R* ^2^ ≤ 4.9%), being a woman (0.1% ≤ *R*^2^ ≤ 4.9%), low education (0.8 ≤ *R*^2^ ≤ 1.8%), being a student (1.3 ≤ *R*^2^ ≤ 3.9%), being an active worker (0.7 ≤ *R*^2^ ≤ 3.2%), cohabitating with more people (0.1 ≤ *R*^2^ ≤ 0.8%), reduced sleep time (0.1 ≤ *R*^2^ ≤ 1.3%), poor sleep quality (2.9 ≤ *R*^2^ ≤ 10.9%), changes in usual sleep patterns (1.2 ≤ *R*^2^ ≤ 2.9%), time spent sitting/laying (0.6 ≤ *R*^2^ ≤ 6.3%), time spent Internet surfing (2.1 ≤ *R*^2^ ≤ 5.4%), eating to cope with distress (0.7 ≤ *R*^2^ ≤ 2.5%), frustration intolerance (3.5 ≤ *R*^2^ ≤ 2 0.2%), and neuroticism (6.5 ≤ *R*^2^ ≤ 15.8%), which were overall associated with poor mood states. On the contrary, exercising (0.3 ≤ *R*^2^ ≤ 5.3%), exercising to cope with distress (0.2 ≤ *R*^2^ ≤ 6.3%), planning daily activities (0.2 ≤ *R*^2^ ≤ 3.8%), extraversion (0.3 ≤ *R*^2^ ≤ 4.9%), openness to experience (0.7 ≤ *R*^2^ ≤ 4.6%), agreeableness (2.0 ≤ *R*^2^ ≤ 12.3%), and conscientiousness (0.8 ≤ *R*^2^ ≤ 3.3%) were associated with better outcomes in at least four out of six mood states. The use of videoconferencing to cope with distress was singular, as it was sometimes associated both with poor (i.e., depression and anxiety) and positive mood states (i.e., vigor and friendliness). 

The remaining variables were less consistently associated with mood states and are not described here in detail due to space limitations (see Table 4). Only a reduced number of variables were not related to any of the mood state (i.e., job loss due to the COVID–19 crisis, number of pets, and cohabitation with a COVID–infected person).

### 3.4. Multivariate Associations with Outcomes

As shown in Table 5, the results obtained with the results of the multivariate analyses were in the same direction as those reported in the univariate regression analysis. In particular, the majority of variables that were consistently and more strongly (larger R^2^) associated with outcomes in a bivariate manner (i.e., age, sleep quality, time spent Internet surfing, frustration intolerance, and neuroticism) were also uniquely associated with several outcomes even after controlling for the contribution of the remaining predictors.

## 4. Discussion

Several calls for mental health investigation during the COVID–19 pandemic have been made and numerous behavioral guidelines have been developed to try to minimize the emotional impact of the current crisis on the population [1,19]. Thus, this study aimed at investigating the emotional impact of the COVID–19 pandemic and included an important comprehensive set of socio–demographic, behavioral, and cognitive correlates of well–being. A key strength of the current study refers to the fact that data was obtained just after the onset of the COVID–19 quarantine in Spain (first day during the evening to the end of the third day of quarantine). As anticipated, the mood of a sample of individuals at quarantine onset was generally poorer compared to a sample of individuals from the general population recruited before the current COVID–19 [17]. Additionally, we generally confirmed the majority of expected associations between socio–demographic, clinical, behavioral, and cognitive/personality factors and individual differences in mood status during the early phase of adaptation to quarantine (first three days).

So far, most studies on the mental health consequences of quarantine and its correlates have been letters to the editor or commentary articles and a reduced number of original investigations have been conducted in China [19]. These studies suggest that the COVID–19 crisis is indeed impacting negatively on the mental well–being of individuals. For example, one study with 1210 Chinese respondents revealed moderate to severe depression, anxiety, and stress in 16.5%, 28.8%, and 8.1% of respondents, respectively [18]. Another investigation during the COVID–19 outbreak in Wuhan (China) reported prevalence rates of depression, anxiety, or both of 48.3%, 22.6%, and 19.4%, respectively [20]. It is important to note that the aforementioned prevalence rates of mental distress do not imply that depression and anxiety problems were a consequence of COVID–19 and could have existed before the crisis. To address this limitation, the present study compared mood levels of participants in the present study with those from past research before the COVID–19 crisis began. 

Our findings support the aforementioned idea that the current crisis or at least quarantine might exert a negative influence on the severity of depressed mood in the general population, although the obtained between–group differences were only small. In relation to anxiety and anger, the results only support the impact of quarantine on the latter. While this is only speculative, it is possible that anxiety, which could have been higher before quarantine due to infection risk, was actually reduced when confined at home (i.e., when risk of infection was low). In fact, note that only 10.7% of respondents perceived that they had a higher–than–average risk of infection. On the contrary, while the onset of quarantine might mitigate the impact of the COVID–19 crisis on anxiety levels, it has been repeatedly shown that quarantine negatively impacts on anger [5], which might be explained by the frustration associated with the consequences of restricted mobility in achieving important personal goals. 

The present study also evidenced that vigor and, to a lesser extent, fatigue, and friendliness might be diminished during the onset of quarantine or as a consequence of the COVID–19 crisis. At first glance, the results on vigor and fatigue might seem contradictory. Of course, it is possible that the sample used for comparison was very fatigued at the time of assessment, which might explain why our sample indicated being less fatigued. However, a distinction between fatigue (e.g., tired, exhausted, and weak) and vigor items (e.g., full of energy, lively, and active) might also shed light on the rationale behind the different direction of findings. In this sense, note that the most frequent activities indicated by individuals in the present study during quarantine onset were largely passive (i.e., sitting/laying, text messaging, Internet surfing, seeking COVID–19 information, and watching TV/series/movies). This, together with the fact that quarantine onset started on a Sunday and imposed resting at home for many participants (note that half of them were not working), might explain why participants in our sample might have been less fatigued, but also less energetic (vigorous). 

One last finding in relation to mood status was that friendliness was lower in our sample than in the comparison group. Friendliness is composed of adjectives like kind, considerate of others, and sympathetic. Thus, the fact that this mood state was low in our sample might be problematic in a situation where pro–social behavior (e.g., respecting governmental recommendations in attempt to decrease the risk of spreading the disease) is of utter importance. While one would expect that this should have been enhanced in the face of the current situation, our results indicate that this mood state might be challenged during quarantine, which could explain why so many antisocial behaviors have occurred since the quarantine onset in Spain (over half a million fines issued and almost 2000 arrests for violating confinement in one month only) [21]. 

Regarding the correlates of well–being, it has been argued that collecting high–quality data during the current COVID–19 crisis is crucial to update existent guidelines [22]. The present study might be an important step in this direction. Overall, our results supported the majority of recommended lifestyles indicated by guidelines and experts (e.g., sleep self–care, exercising, planning some daily activities, and minimizing the time spent seeking COVID–19 information and emotional eating) [6,7,8,9,10,11,12]. We also replicated the results from a study in China indicating that students and women were at higher risk for low mood [18]. Overall, however, the contribution of these variables on mood states was modest (generally less than 5% explained variance by a single variable). Exceptions to this were cognitive/personality characteristics, namely frustration intolerance and neuroticism, which explained up to 20.2% and 15.8%, respectively, of mood states. 

On the one hand, frustration intolerance refers to an inability to acknowledge (“cannot stand”) the existence of discomfort in life [23]. In particular, the intolerance of emotions component taps into the difficulties in accepting difficult thoughts and feelings [15]. On the other hand, neuroticism is a personality trait characterized by a tendency to experience negative emotions in the face of threat, loss, or frustration [24]. Both psychological factors have been consistently associated with poor mental and often physical health status of individuals and argued to predispose individuals to psychopathology [25,26]. In the light of the previous and the large explained variance obtained in the present study, both frustration intolerance and neuroticism might be important factors to be considered to detect vulnerable individuals to quarantine onset. While changing neuroticism might require more structured interventions, one straightforward recommendation for the general population in the direction of increasing tolerance of emotions might be to acknowledge difficult emotions as part of the current situation (i.e., quarantine) without trying to get rid of them (e.g., by eating emotionally or seeking COVID–19 information excessively).

The present study limitations include the focus on cross–sectional data only, the limited number of days (i.e., only three), and the fact that analyses are based on linear regressions, which poses the question of whether we are leaving out of sight possible non–linear or interaction effects. However, none of the guidelines take non–linearity into account when making their recommendations, but this exceeds the scope of this paper and should be addressed in future studies. Additionally, answers from three consecutive days were collapsed, which prevents us from exploring whether the correlation between study variables differed across days. This was done to reduce the already large number of statistical comparisons and because measures were obtained in three consecutive days as opposed to very different moments during quarantine. However, an important question for future research might be to explore whether the importance of correlates of mood remains unchanged or not at different stages during quarantine (e.g., whether frustration tolerance is not only important at the beginning of quarantine but also when individuals have been under quarantine for several weeks). Note also that data from the general public used to compare average mood in the present study was obtained in 2013 and in a single region of Spain. Therefore, it is possible that the differences in time and place of data collection might have affected the results. In relation to generalizability of findings, it is also important to note that, despite answers were obtained from almost 2700 participants from all 17 regions in Spain and the proportion of responses per region were consistent with the population numbers per region, the overrepresentation of females and young adults in the sample should be taken into account when considering the generalizability of the results. Finally, it is important to consider that in the study we present correlates between several socio–demographic, behavioral, and personality/cognitive factors and mood status during the first three days of quarantine only. Thus, because the strength of the obtained correlations was not compared against data before the quarantine or before the COVID–19 crisis we cannot assume that the correlations found are due to quarantine or the COVID–19 crisis. Therefore, it is possible that the correlations found are a general phenomenon rather than a consequence of confinement or the COVID–10 crisis.

## 5. Conclusions

While acknowledging the previous shortcomings, the present study might as well add to the existing literature on the impact of the COVID–19 crisis or quarantine on well–being and its correlates. An important finding was that the majority of recommendations made by renowned institutions and experts for adaptation to quarantine were supported, even though not all of them might be related to all mood states or, at least, to the same extent. In this sense, the study pointed to frustration intolerance and neuroticism as the most important vulnerability factors for low mood during quarantine onset. Additionally and consistent with guidelines, behaviors like having a good quality of sleep, exercising, and planning daily activities were found to be associated with better mood, while the opposite relationship was found with spending a lot of time Internet surfing, seeking coronavirus–related information, and sitting/lying for long periods. Also, interestingly, a novel finding was that younger participants (young adults) presented poorer mood status. As a final remark, it is important to note the contribution of the majority of these variables on outcomes, particularly age, sleep quality, Internet surfing, videoconferencing, frustration intolerance, and neuroticism, remained significant even after controlling for the role of all the other factors. This suggests that the previous variables might be relevant risk/protective factors to be considered together for prevention and treatment purposes. Ultimately, these results might be important to guide psychological interventions in the face of the COVID–19 in a more effective way, which is crucial in the current situation [13].

## Figures and Tables

**Table 1:** Behavioral characteristics of the sample (in the past 24 h).

Frequency of Daily Behaviors	*n* (%)	Range
Sitting/laying		0–4
Never	32 (1.2)	
Rarely	543 (20.3)	
Occasionally	918 (34.3)	
A moderate amount	689 (25.7)	
A great deal	498 (18.6)	
Text messaging		0–6
None	42 (1.6)	
1–5 persons	547 (20.4)	
6–10 persons	748 (28.0)	
11–15 persons	504 (18.8)	
16–20 persons	289 (10.8)	
21–25 persons	113 (4.2)	
>25 persons	433 (16.2)	
Internet surfing		0–7
None	62 (2.3)	
≤ 30’	191 (7.2)	
30’–60’	452 (16.9)	
60’–90’	399 (14.9)	
90’–120’	321 (12.0)	
2–3 h	540 (20.2)	
4–5 h	328 (12.3)	
>5 h	377 (14.1)	
Sunlight exposure		0–7
None	508 (19.0)	
≤30’	907 (33.9)	
30’–60’	597 (22.3)	
60’–90’	267 (10.0)	
90’–120’	127 (4.7)	
2–3 h	124 (4.6)	
4–5 h	57 (2.1)	
> 5 h	91 (3.4)	
COVID information		0–8
None	150 (5.6)	
≤30’	523 (19.5)	
30’–60’	731 (27.3)	
60’–90’	445 (16.6)	
90’–20’	299 (11.2)	
2–3 h	293 (10.9)	
4–5 h	124 (4.6)	
>5 h	114 (4.3)	
TV/series/movies		0–7
None	115 (4.3)	
≤30’	157 (5.9)	
30’–60’	303 (11.3)	
60’–90’	391 (14.6)	
90’–120’	451 (16.8)	
2–3 h	594 (22.2)	
4–5 h	396 (14.8)	
>5 h	272 (10.2)	

**Table 2:** Coping with boredom/distress characteristics of the sample (in the past 24 h).

Frequency of Coping Strategies	*n* (%)	Range
Watch TV		0–4
Not at all	305 (11.4)	
Slightly	612 (22.9)	
Moderately	608 (22.8)	
Very much	808 (30.3)	
Extremely	334 (12.5)	
Work/study		0–4
Not at all	1018 (38.3)	
Slightly	550 (20.7)	
Moderately	447 (16.8)	
Very much	503 (18.9)	
Extremely	142 (5.3)	
Videoconferences		0–4
Not at all	1048 (39.4)	
Slightly	679 (25.5)	
Moderately	478 (18.0)	
Very much	390 (14.7)	
Extremely	66 (2.5)	
Exercising		0–4
Not at all	1502 (56.6)	
Slightly	561 (21.1)	
Moderately	350 (13.2)	
Very much	24 (8.4)	
Extremely	19 (0.7)	
Cleaning/tidying		0–4
Not at all	359 (13.5)	
Slightly	755 (28.4)	
Moderately	676 (25.4)	
Very much	745 (28.0)	
Extremely	126 (4.7)	
Eating		0–4
Not at all	41 (1.5)	
Slightly	368 (13.8)	
Moderately	1573 (59.1)	
Very much	596 (22.4)	
Extremely	83 (3.1)	
Play games with others		0–4
Not at all	1368 (51.3)	
Slightly	435 (16.3)	
Moderately	335 (12.6)	
Very much	431 (16.2)	
Extremely	97 (3.6)	

**Table 3:** Means and standard deviations of mood states and mean–differences with scores from the general population before the COVID–19 epidemic.

Mood States	Current Sample Mean (*SD*)	General Population Mean (*SD*)	*t* (Cohen’s *d*)	95% CI
Depression	4.47 (4.09)	3.25 (3.91)	5.79 * (0.30)	0.81, 1.63
Anxiety	4.94 (4.57)	5.01 (4.54)	–0.29 (0.02)	−0.55, 0.41
Anger	3.90 (3.96)	2.93 (3.84)	4.81 * (0.25)	0.57, 1.38
Vigor	7.50 (4.27)	10.48 (4.48)	–12.49 * (0.69)	−3.45, −2.51
Fatigue	4.09 (3.96)	5.45 (4.48)	–5.75 * (0.34)	−1.83, −0.89
Friendliness	11.74 (3.73)	13.46 (3.36)	–9.41 * (0.47)	−2.08, −1.36

* *p* < 0.001.

**Table 4:** Univariate linear regression analyses.

Study Predictors	Depression		Anxiety		Anger		Vigor		Fatigue		Friendliness	
	*R* ^2^	B (95% CI)	*R* ^2^	B (95% CI)	*R* ^2^	B (95% CI)	*R* ^2^	B (95% CI)	*R* ^2^	B (95% CI)	*R* ^2^	B (95% CI)
Socio−demographic and clinical												
Age ^c^	**0.049**	−0.07 (−0.08, −0.06) *	**0.039**	−0.07 (−0.08, −0.05) *	**0.034**	−0.06 (−0.07, −0.04) *	**0.041**	0.06 (0.05, 0.08) *	**0.039**	−0.06 (−0.07, −0.05) *	**0.039**	−0.06 (−0.07, −0.05) *
Gender ^a^	**0.011**	1.01 (0.64, 1.38) *	**0.013**	1.24 (0.82, 1.65) *	0.001	0.37 (−0.01, 0.75)	**0.026**	−1.64 (−2.02, −1.26) *	**0.008**	0.85 (0.49, 1.21) *	<0.001	0.10 (−0.24, 0.44)
In a relationship ^a^	**0.013**	−0.98 (−1.30, −0.65) *	<0.001	0.08 (−0.28, 0.45)	0.001	−0.24 (−0.57, 0.09)	0.002	0.35 (0.01, 0.69)	0.001	−0.31 (−0.63, <0.01)	0.001	0.28 (−0.02, 0.57)
Education ^a^	**0.018**	−1.13 (−1.45, −0.81) *	**0.008**	−0.87 (−1.22, −0.51) *	**0.017**	−1.13 (−1.45, −0.81) *	**0.007**	0.72 (0.39, 1.06) *	**0.011**	−0.86 (−1.17, −0.55) *	**0.015**	0.95 (0.66, 1.24) *
Home income (month) ^b^	**0.014**	−.25 (−0.33, −0.17) *	<0.001	−0.05 (−0.14, 0.05)	0.001	−0.08 (−0.17, <0.01)	**0.014**	0.26 (0.17, 0.35) *	0.004	−0.13 (−0.21, −0.05)	0.001	0.07 (−0.01, 0.14)
Lost job ^a^	0.002	0.38 (0.11, 0.75)	0.002	0.53 (0.11, 0.94)	0.001	0.34 (−0.34, 0.71)	<0.001	−0.16 (−0.54, 0.23)	<0.001	<0.01 (−0.36, 0.36)	<0.001	−0.01 (−0.35, 0.32)
Student ^a^	**0.039**	1.81 (1.47, 2.15) *	**0.018**	1.37 (0.98, 1.75) *	**0.026**	1.50 (1.15, 1.85) *	**0.013**	−1.09 (−1.44, −0.73) *	**0.023**	1.32 (1.00, 1.66) *	**0.017**	−1.08 (−1.39, −0.76)
Works ^a^	**0.032**	1.46 (1.16, 1.77)	**0.007**	0.78 (0.43, 1.13) *	**0.018**	1.12 (0.80, 1.43) *	**0.005**	−0.59 (−0.91, −0.26) *	**0.012**	0.89 (0.59, 1.19) *	**0.010**	−0.75 (−1.03, −0.46) *
House size ^b^	**0.007**	−0.23 (−0.33, −0.13) *	0.001	−0.07 (−0.19, 0.04)	0.001	−0.09 (−0.19, 0.01)	**0.014**	0.33 (0.22, 0.43) *	**0.004**	−0.17 (−0.27, −0.07) *	0.001	0.07 (−0.03, 0.16)
Pets ^a^	0.001	0.29 (−0.02, 0.60)	0.002	0.45 (0.10, 0.78)	0.003	0.46 (0.15, 0.78)	0.003	−0.44 (−0.77, −0.12)	**0.004**	0.51 (0.21, 0.81) *	0.003	−0.43 (−0.72, 0.15)
Pets−number ^c^	0.001	0.10 (−0.04, 0.24)	0.001	0.10 (−0.05, 0.25)	0.002	0.17 (0.03, 0.31)	0.002	−0.16 (−0.30, −0.01)	0.003	0.20 (0.07, 0.33)	0.003	−0.18 (−0.30, −0.05)
Cohabitation ^a^	**0.007**	−1.24 (−1.78, −0.69) *	0.005	1.15 (0.54, 1.76) *	0.003	0.78 (0.23, 1.34)	0.002	0.61 (0.04, 1.18)	0.001	0.46 (−0.73, 0.98)	0.001	−0.45 (−0.95, 0.05)
Cohabitation−number ^c^	0.001	−0.08 (−0.20, 0.03)	**0.005**	0.23 (0.10, 0.36) *	**0.006**	0.24 (0.12, 0.36) *	<0.001	−0.06 (−0.18, 0.06)	**0.007**	0.24 (0.13, 0.35) *	**0.008**	−0.24 (−0.35, −0.14) *
Cohabitation children ^a^	**0.029**	−1.54 (−1.88, −1.20) *	0.004	−0.62 (−1.00, −0.24)	**0.005**	−0.62 (−0.96, −0.27) *	0.003	0.49 (0.14, 0.85)	0.002	−0.40 (−0.73, −0.07)	0.001	0.31 (−0.01, 0.62)
Cohabitation infected ^a^	<0.001	0.40 (−1.32, 2.12)	<0.001	−0.05 (−1.96, 1.87)	<0.001	−0.35 (−2.08, 1.39)	0.001	−1.10 (−2.89, 0.69)	0.001	1.01 (−0.65, 2.68)	<0.001	−0.43 (−2.00, 1.14)
COVID−19 infection risk ^b^	<0.001	0.04 (−0.06, 0.14)	**0.005**	0.21 (0.10, 0.31) *	0.003	0.15 (0.05, 0.25)	<0.001	−0.02 (−0.13, 0.08)	0.001	0.06 (−0.03, 0.16)	0.002	−0.10 (−0.19, −0.02)
Vulnerable relative ^a^	**0.004**	0.57 (0.21, 0.92)	**0.009**	0.98 (0.58, 1.37) *	0.003	0.55 (0.20, 0.91)	0.001	−0.24 (−0.61, 0.13)	0.004	0.55 (0.21, 0.89)	0.001	−0.27 (−0.59, 0.05)
Use of psychotropic drugs	**0.004**	2.00 (0.84, 3.15) *	0.003	1.77 (0.48, 3.06)	0.001	1.08 (−0.09, 2.25)	0.003	−1.80 (−3.01, −0.60)	0.003	1.57 (0.46, 2.69)	0.001	−0.65 (−1.71, 0.40)
Behavioral												
Sleep h ^c^	**0.005**	−0.24 (−0.36, −0.12) *	**0.007**	−0.30 (−0.44,−0.16) *	**0.005**	−0.23 (−0.36, −0.11) *	<0.001	0.02 (−0.11, 0.15)	**0.013**	−0.37 (−0.49, −0.25) *	<0.003	−0.06 (−0.17, 0.06)
Sleep quality ^b^	**0.084**	−0.87 (−0.98, −0.77) *	**0.096**	−1.04 (−1.16, −0.92) *	**0.071**	−0.81 (−0.92, −0.70) *	**0.064**	0.79 (0.68, 0.91) *	**0.109**	−0.96 (−1.06, −0.86) *	**0.029**	0.47 (0.37, 0.57) *
Sleep pattern−usual ^a^	**0.023**	−1.25 (−1.56, −0.94) *	**0.012**	−0.99 (−1.33, −0.64) *	**0.017**	−1.07 (−1.39, −0.76) *	**0.029**	1.46 (1.14, 1.78) *	**0.017**	−1.04 (−1.34, −0.74) *	**0.018**	1.01 (0.73, 1.29) *
Frequency−Interactions (in person) ^c^	<0.001	0.05 (−0.04, 0.13)	**0.004**	0.16 (0.07, 0.26) *	0.004	0.15 (0.06, 0.23)	<0.001	−0.01 (−0.10, 0.09)	0.002	0.10 (0.02, 0.18)	0.002	−0.10 (−0.18, −0.02)
Frequency−Interactions (virtual) ^c^	<0.001	0.02 (−0.06, 0.10)	0.003	0.13 (0.04, 0.21)	<0.001	0.04 (−0.04, 0.12)	**0.009**	0.20 (0.12, 0.29) *	<0.001	−0.01 (−0.09, 0.07)	**0.006**	0.15 (0.08, 0.22) *
Frequency−Sitting/laying ^b^	**0.040**	0.78 (0.64, 0.93) *	**0.006**	0.34 (0.17, 0.50) *	**0.019**	0.55 (0.40, 0.70) *	**0.063**	−1.18 (−1.18, −0.89) *	**0.025**	0.60 (0.46, 0.75) *	**0.020**	−0.51 (−0.64, −0.37) *
Frequency−Text messaging ^b^	0.003	−0.13 (−0.22, −0.04)	<0.001	−0.01 (−0.10, 0.10)	<0.001	−0.02 (−0.11, 0.07)	**0.015**	0.30 (0.21, 0.40) *	**0.005**	−0.16 (−0.25, −0.07) *	**0.024**	0.34 (0.26, 0.42) *
Frequency−Internet surfing ^b^	**0.054**	0.49 (0.41, 0.56) *	**0.031**	0.41 (0.33, 0.50) *	**0.046**	0.45 (0.38, 0.53) *	**0.043**	−0.46 (−0.54, −0.37) *	**0.040**	0.41 (0.33, 0.48) *	**0.021**	−0.28 (−0.35, −0.21) *
Frequency−Sunlight exposure ^b^	**0.012**	−0.26 (−0.35, −0.17) *	0.002	−0.13 (−0.23, −0.03)	0.003	−0.13 (−0.23, −0.04)	**0.022**	0.36 (0.27, 0.46) *	**0.011**	−0.24 (−0.32, −0.15) *	0.003	0.12 (0.04, 0.20)
Frequency−COVID information ^b^	0.004	0.12 (0.05, 0.20)	**0.018**	0.31 (0.23, 0.40) *	**0.004**	0.13 (0.05, 0.21) *	0.003	−0.13 (−0.21, −0.04)	0.003	0.11 (0.03, 0.18)	0.003	0.10 (0.03, 0.17)
Frequency−TV/series/movies ^b^	**0.004**	0.14 (0.06, 0.22) *	0.001	0.08 (−0.01, 0.17)	**0.004**	0.15 (0.06, 0.23) *	**0.007**	−0.19 (−0.27, −0.10) *	0.001	0.05 (−0.03, 0.13)	0.001	−0.07 (−0.15, <0.01)
Frequency−Exercising ^b^	**0.006**	−0.27 (−0.40, −0.14) *	0.002	−0.18 (−0.33, −0.04)	0.004	−0.22 (−0.35, −0.08)	**0.056**	0.86 (0.73, 0.99) *	**0.011**	−0.35 (−0.48, −0.23) *	**0.013**	0.37 (0.25, 0.48) *
Exercised ^a^	**0.007**	−0.70 (−1.01, −0.39) *	0.003	−0.53 (−0.87, −0.18)	**0.004**	−0.65 (−0.96, −0.33)	**0.053**	1.97 (1.66, 2.29) *	**0.010**	−0.80 (−1.09, −0.50) *	**0.015**	0.91 (0.63, 1.20) *
Coping−Watch TV ^b^	**0.005**	0.24 (0.11, 0.37) *	0.003	0.21 (0.07, 0.36)	**0.007**	0.29 (0.16, 0.42) *	**0.007**	−0.30 (−0.43, −0.16) *	0.003	0.17 (0.05, 0.29)	<0.001	0.01 (−0.10, 0.13)
Coping−Work/study ^b^	**0.005**	−0.23 (−0.35, −0.11) *	0.001	−0.10 (−0.24, 0.03)	0.002	−0.15 (−0.27, −0.03)	**0.029**	0.56 (0.44, 0.68) *	0.002	−0.14 (−0.26, −0.03)	**0.006**	0.22 (0.11, 0.33) *
Coping−Videoconferences ^b^	**0.005**	0.25 (0.11, 0.38) *	**0.011**	0.41 (0.26, 0.56) *	0.003	0.19 (0.05, 0.32) *	**0.007**	0.30 (0.16, 0.44) *	0.002	0.16 (0.03, 0.29)	**0.008**	0.29 (0.17, 0.41) *
Coping−Exercising ^b^	**0.006**	−0.30 (−0.45, −0.15) *	0.002	−0.18 (−0.35, −0.01)	**0.005**	−0.29 (−0.44, −0.13) *	**0.063**	1.05 (0.89, 1.20) *	**0.009**	−0.37 (−0.52, −0.23) *	**0.013**	0.42 (0.28, 0.56) *
Coping−Cleaning/tidying ^b^	0.003	−0.19 (−0.32, −0.05)	<0.001	−0.01 (−0.16, 0.15)	<0.001	−0.06 (−0.20, 0.09)	**0.010**	0.38 (0.23, 0.52) *	0.002	−0.15 (−0.28, −0.02)	**0.007**	0.28 (0.16, 0.41) *
Coping−Eating ^b^	**0.013**	0.63 (0.42, 0.85) *	**0.018**	0.84 (0.60, 1.07) *	**0.025**	0.90 (0.69, 1.11) *	**0.007**	−0.48 (−0.70, −0.25) *	**0.017**	0.71 (0.51, 0.91) *	**0.010**	−0.52 (−0.71, −0.32) *
Coping−Play games with others ^b^	**0.006**	−0.25 (−0.37, −0.12) *	<0.001	0.07 (−0.06, 0.21)	<0.001	−0.03 (−0.15, 0.10)	**0.007**	0.29 (0.16, 0.42) *	<0.001	−0.03 (−0.15, 0.09)	**0.005**	0.21 (0.10, 0.32) *
Planned daily activities ^b^	**0.009**	−0.36 (−0.50, −0.22) *	0.002	−0.20 (−0.36, 0.01)	**0.005**	−0.26 (−0.41, −0.12) *	**0.038**	0.75 (0.61, 0.90) *	**0.005**	−0.24 (−0.38, −0.11) *	**0.020**	0.48 (0.35, 0.61) *
Personality/cognitive												
Frustration intolerance ^c^	**0.202**	0.27 (0.25, 0.29) *	**0.143**	0.26 (0.23, 0.28) *	**0.155**	0.24 (0.21, 0.26) *	**0.049**	−0.14 (−0.16, −0.12) *	**0.100**	0.18 (0.16, 0.21) *	**0.035**	−0.11 (−0.12, −0.08) *
Neuroticism ^c^	**0.154**	0.55 (0.50, 0.60) *	**0.158**	0.62 (0.57, 0.68) *	**0.141**	0.53 (0.48, 0.58) *	**0.078**	−0.41 (−0.46, −0..35) *	**0.093**	0.41 (0.36, 0.46) *	**0.065**	−0.32 (−0.37, −0.28) *
Extraversion ^c^	**0.017**	−0.18 (−0.24, −0.13) *	0.004	−0.10 (−0.16, −0.04)	**0.007**	−0.12 (−0.17, −0.06) *	**0.037**	0.28 (0.22, 0.33) *	**0.019**	−0.19 (−0.24, −0.14) *	**0.049**	0.28 (0.23, 0.33) *
Openness to experience ^c^	**0.010**	−0.17 (−0.23, −0.10) *	**0.007**	−0.16 (−0.23, −0.08) *	**0.008**	−0.15 (−0.22, −0.08) *	**0.038**	0.35 (0.28, 0.41) *	**0.011**	−0.18 (−0.24, −0.11) *	**0.046**	0.33 (0.27, 0.39) *
Agreeableness ^c^	**0.049**	−0.42 (−0.49, −0.35) *	**0.045**	−0.45 (−0.53, −0.37)	**0.117**	−0.65 (−0.72, −0.58) *	**0.020**	0.285 (0.20, 0.35) *	**0.028**	−0.31 (−0.38, −0.24) *	**0.123**	0.60 (0.54, 0.67) *
Conscientiousness ^c^	**0.018**	−0.21 (−0.27, −0.15) *	**0.008**	−0.16 (−0.23, −0.09) *	**0.019**	−0.22 (−0.28, −0.16) *	**0.033**	0.30 (0.24, 0.36) *	**0.022**	−0.23 (−0.29, −0.17) *	**0.030**	0.25 (0.19, 0.30) *

^a^ categorical (*dichotomous*); ^b^ categorical (ordinal); ^c^ continuous. In all dichotomous variables, except gender and education, the reference value is “yes”. For gender and education, the reference values are “female” and “≥12 years”, respectively. Among all categorical, non–dichotomous variables, only “exercising” was dichotomized because an analysis of differences in outcome scores across categories suggested that a linear association was adequately represented the relationship between the remaining ordinal variables and outcomes. * *p* < 0.001; In the *R*^2^ columns, significant *R*^2^ estimates at *p* < 0.001 are presented in bold.

**Table 5:** Multivariate linear regression analyses using backward elimination.

Study Predictors	Depression		Anxiety		Anger		Vigor		Fatigue		Friendliness	
	*R* ^2^	B (95% CI)	*R* ^2^	B (95% CI)	*R* ^2^	B (95% CI)	*R* ^2^	B (95% CI)	*R* ^2^	B (95% CI)	*R* ^2^	B (95% CI)
	0.343		0.309		0.299		0.301		0.246		0.245	
Socio−demographic and clinical												
Age ^c^		−0.04 (−0.05, −0.02) *		−0.05 (−0.06, −0.03) *		−0.03 (−0.04, −0.01) *		0.04 (0.03, 0.06) *		−0.04 (−0.05, −0.02) *		0.02 (<0.01, 0.03)
Gender ^a^								−1.37 (−1.74, −1.01) *				
In a relationship ^a^				0.42 (0.09, 0.76)				−0.39 (0.73, −0.05)				
Home income (month) ^b^				0.10 (0.01, 0.18)								
Lost job ^a^				0.35 (−0.01,0.72)								
Works ^a^		0.42 (0.13, 0.71)										
Pets−number ^c^												
Cohabitation ^a^		−1.53 (−2.03, −1.04) *						1.25 (0.68, 1.81) *				
Cohabitation children ^a^								−0.69 (−1.09, 0.29)		0.45 (0.10, 0.79)		−0.47 (−0.81, −0.12)
Vulnerable relative ^a^		0.31 (−0.01, 0.62)		0.64 (0.28, 0.99) *								
Use of psychotropic drugs								−1.12 (−2.22, −0.03)				
Behavioral												
Sleep h ^c^		−0.12 (−0.24, 0.01)				−0.11 (−0.23, 0.02)				−0.17 (−0.30, −0.05)		−0.12 (−0.23, <0.01)
Sleep quality ^b^		−0.52 (−0.63, −0.41) *		−0.69 (−0.81, −0.57) *		−0.48 (−0.60, −0.36) *		0.53 (0.42, 0.65) *		−0.66 (−0.78, −0.55) *		0.33 (0.22, 0.44) *
Sleep pattern−usual ^a^		−0.26 (−0.54, 0.02)						0.47 (0.16, 0.78)				0.38 (0.10, 0.66)
Frequency−Sitting/laying ^b^		0.22 (0.08, 0.36)						−0.43 (−0.59, −0.27) *		0.21 (0.06, 0.36)		−0.14 (−0.28, 0.01)
Frequency−Text messaging ^b^								0.13 (0.04, 0.22)				0.17 (0.09, 0.26) *
Frequency−Internet surfing ^b^		0.14 (0.05, 0.22)				0.17 (0.09, 0.25) *		−0.15 (−0.24, −0.06) *		0.14 (0.06, 0.23)		−0.14 (−0.22, −0.06) *
Frequency−Sunlight exposure ^b^		−0.11 (−0.19, −0.03)						0.11 (0.02, 0.20)		−0.13 (−0.21, −0.04)		
Frequency−COVID information ^b^		0.13 (0.05, 0.20) *		0.35 (0.27, 0.43) *		0.09 (0.01, 0.17)		−0.10 (−0.18, −0.02)		0.10 (0.03, 0.18)		0.09 (0.02, 0.17)
Frequency−TV/series/movies ^b^										−0.10 (−0.20, −0.01)		
Frequency−Exercising ^b^												0.13 (0.01, 0.25)
Exercised ^a^								0.17 (<0.01, 0.34)				
Coping−Watch TV ^b^						0.14 (0.02, 0.26)				0.13 (−0.02, 0.27)		0.11 (−0.01, 0.23)
Coping−Work/study ^b^								0.28 (0.16, 0.40) *				
Coping−Videoconferences ^b^		0.10 (−0.02, −0.05)		0.26 (0.12, 0.39) *				0.26 (0.12, 0.39) *				0.22 (0.10, 0.34) *
Coping−Exercising ^b^								0.39 (0.20, 0.58) *				
Coping−Cleaning/tidying ^b^								0.12 (−0.02, 0.26)				
Coping−Eating ^b^				0.33 (0.11, 0.54)		0.42 (0.22, 0.62) *				0.29 (0.09, 0.49)		−0.23 (−0.42, −0.05) *
Coping−Play games with others ^b^								0.21 (0.09, 0.34)				
Planned daily activities ^b^				0.12 (−0.02, 0.27)				0.22 (0.08, 0.36)				0.21 (0.08, 0.33)
Personality/cognitive												
Frustration intolerance ^c^		0.17 (0.15, 0.19) *		0.15 (0.13, 0.18) *		0.15 (0.12, 0.17) *		−0.03 (−0.06, −0.01)		0.10 (0.08, 0.12) *		−0.02 (−0.04, −0.01)
Neuroticism ^c^		0.22 (0.16, 0.28) *		0.36 (0.30, 0.42) *		0.16 (0.10, 0.22) *		−0.12 (−0.18, −0.07) *		0.16 (0.11, 0.22) *		
Extraversion ^c^								0.08 (0.02, 0.13)		−0.07 (−0.11, −0.02)		0.13 (0.08, 0.18) *
Openness to experience ^c^								0.17 (0.10, 0.23) *				0.14 (0.08, 0.20) *
Agreeableness ^c^		−0.09 (−0.16, −0.02)				−0.38 (−0.45, −0.31) *						0.48 (0.42, 0.55) *
Conscientiousness ^c^								0.07 (0.01, 0.13)		−0.06 (−0.12, −0.01)		

^a^ categorical (*dichotomous*); ^b^ categorical (ordinal); ^c^ continuous. In all dichotomous variables, except gender and education, the reference value is “yes”. For gender, the reference value is “female”. Among all categorical, non–dichotomous variables, only “exercising” was dichotomized because an analysis of differences in outcome scores across categories suggested that a linear association was adequately represented the relationship between the remaining ordinal variables and outcomes. * *p* < 0.001; The *R*^2^ estimates are from the final model with the predictors that were retained after the backward elimination procedure. Only variables that were retained for at least one predictor are included in the Table for readability reasons.
